# Rapid Diagnostic Test for Hepatitis B Virus Viral Load Based on Recombinase Polymerase Amplification Combined with a Lateral Flow Read-Out

**DOI:** 10.3390/diagnostics12030621

**Published:** 2022-03-02

**Authors:** Charly Mayran, Vincent Foulongne, Philippe Van de Perre, Chantal Fournier-Wirth, Jean-Pierre Molès, Jean-François Cantaloube

**Affiliations:** Pathogénèse et Contrôle des Infections Chroniques et Emergentes, Université de Montpellier, Etablissement Français du Sang, Inserm, Université des Antilles, 34184 Montpellier, France; charly.mayran@efs.sante.fr (C.M.); v-foulongne@chu-montpellier.fr (V.F.); p-van_de_perre@chu-montpellier.fr (P.V.d.P.); chantal.fournier@efs.sante.fr (C.F.-W.); jean-pierre.moles@inserm.fr (J.-P.M.)

**Keywords:** hepatitis B virus, mother to child transmission, Chelex extraction, recombinase polymerase amplification, lateral flow, immunochromatographic strip

## Abstract

Hepatitis B (HBV) infection is a major public health concern. Perinatal transmission of HBV from mother to child represents the main mode of transmission. Despite the existence of effective immunoprophylaxis, the preventive strategy is inefficient in neonates born to mothers with HBV viral loads above 2 × 10^5^ IU/mL. To prevent mother-to-child transmission, it is important to identify highly viremic pregnant women and initiate antiviral therapy to decrease their viral load. We developed a simple innovative molecular approach avoiding the use of automatic devices to screen highly viremic pregnant women. This method includes rapid DNA extraction coupled with an isothermal recombinase polymerase amplification (RPA) combined with direct visual detection on a lateral flow assay (LFA). We applied our RPA-LFA approach to HBV DNA-positive plasma samples with various loads and genotypes. We designed a triage test by adapting the analytical sensitivity to the recommended therapeutic decision threshold of 2 × 10^5^ IU/mL. The sensitivity and specificity were 98.6% (95% CI: 92.7–99.9%) and 88.2% (95% CI: 73.4–95.3%), respectively. This assay performed excellently, with an area under the ROC curve value of 0.99 (95% CI: 0.99–1.00, *p* < 0.001). This simple method will open new perspectives in the development of point-of-care testing to prevent HBV perinatal transmission.

## 1. Introduction

Prevalence of hepatitis B (HBV) infection ranges worldwide from 0.5% to more than 7% in the WHO-defined African region [1,2]. The main long-term complications associated with chronic HBV infection are liver cirrhosis and hepatocarcinoma [2,3]. Deaths due to these complications increased by more than 33% between 1990 and 2013, with 821,000 deaths worldwide in 2019 [4].

To reduce incidence of mother-to-child HBV transmission (HBV MTCT), WHO recommends administration of HBV vaccine to all neonates and, in addition, recommends administration of immunoglobulins against HBV (HBIG) to neonates born to women infected with HBV within 12 h of delivery. The major risk of failure of this treatment is the presence of a high viral load in the pregnant woman [5,6,7,8,9,10]. Prevalence of HBs antigen positive (HBsAg+) pregnant women ranges from 3.2% in Eritrea, 4.8% in Burkina Faso to 11% in Republic of South Soudan [11,12]. Among them, about 7.5% in Burkina Faso [11], 5.6% in the Democratic Republic of the Congo [7], and 5.2% in Mozambique [13] have an elevated viral load. However, administrating the birth dose of HBV vaccine is frequently logistically difficult, making the HBV MTCT prevention program suboptimal. To overcome these difficulties, the European Association for the Study of the Liver [3] and the American Association for the Study of Liver Diseases [14] recommend initiating antiviral therapy in pregnant women with HBV viral loads greater than 2 × 10^5^ IU/mL between 28 weeks and 32 weeks of gestation [5] to prevent mother-to-child transmission. African countries such as Burkina Faso have initiated programs including treatment of highly viremic mothers to strengthen the prevention of HBV MTCT [11]. In addition, other countries were engaged in this way, performing feasibility study for arresting vertical transmission of HBV [7] or piloting an intervention of the prevention of HBV MTCT [13]. This therefore requires knowledge of the HBV viral loads of women.

HBV viral load is measured using commercialized system such as the Xpert^®^HBV Viral Load Test (Cepheid, Sunnyvale, CA, USA) or the COBAS^®^ Ampliprep/COBAS^®^ TaqMan^®^ system (Roche Diagnostics, Indianapolis, IN, USA) [15]. Others kits are also based on a quantitative real-time polymerase chain reaction (PCR) assay [16]. However, these technologies are poorly accessible in low- and middle-income countries (LMICs), as PCR requires well-equipped laboratories including the use of sophisticated instruments and trained personnel. Costs of these technologies will be prohibitive in the healthcare systems of some countries and therefore will not be available to a large majority of women. In Burkina Faso, the cost of HBsAg testing (USD 3.88) and HBV DNA quantification (USD 37.02) is paid by the patient [11]. There is therefore a great need to facilitate access to these molecular analyzes thanks to point-of-care tests at lower costs and usable in a simple medical center.

In recent years, several methods alternative to PCR have been developed for amplifying nucleic acids outside molecular biology laboratories [17,18,19,20]. These tests are mostly based on isothermal amplifications, avoiding the use of sophisticated thermal cyclers [17,18,21]. Among them, recombinase polymerase amplification (RPA) [17,21,22] has been used for the detection of many pathogens, such as SARS-CoV-2 [23,24,25] and HIV [26]. Briefly, a recombinase facilitates insertion of primers into the DNA at a relatively low and constant temperature between 37 °C and 42 °C. Single-stranded DNA binding proteins stabilize the displaced DNA strand. Then, a polymerase extends primers to synthesize a new antisense DNA strand. As with PCR, the use of two opposing primers allows the exponential amplification of the target sequence.

Recent publications have reported the development of HBV nucleic acid isothermal amplification assays. The detection steps involve either fluorescence [27,28,29] or electrochemical read-outs [30]. All these assays are qualitative. The aim of our work was to develop a semi-quantitative assay that could accurately detect HBV infection in pregnant women with viral loads of 2 × 10^5^ IU/mL and above. Furthermore, to move closer to a point-of-care format, we tested whether the amplified products could be visually detected on a lateral flow strip. We then evaluated the performance of this method on HBV-positive and HBV-negative plasma samples.

## 2. Materials and Methods

### 2.1. Plasma Samples

A total of 89 plasma samples (Supplementary Table S1) from blood donors previously screened for HBV DNA by routine nucleic acid testing were obtained from the Etablissement Français du Sang (EFS, Saint-Denis, France). These samples were genotyped and titrated as previously described [31]. The panel included samples from HBV genotypes A (n = 27), B (n = 11), C (n = 7), D (n = 31), E (n = 11), and F (n = 1), and one undefined sample (n = 1).

For the selection of primers and analysis of the detection limit of our assay, we used, as the HBV standard, a genotype D HBV plasma sample titrated at a viral load of 1.46 × 10^6^ IU/mL. Plasma samples from blood donors shown by PCR to be HBV negative (n = 19) were collected by the EFS and thereafter used as negative controls. We used one HIV-positive and one HCV-positive sample collected by the EFS as controls for specificity. All donors signed an informed consent form. All samples were stored at −80 °C under the collection number DC-2021-4414.

### 2.2. Primers, Probe, and Internal Control

The primers selected to conduct the RPA reaction were based on previous reports by Shen et al. [29] and Yi et al. [32] after checking for accuracy against a large set of HBV strains. The HBV-Fc forward primer sequence was consequently modified from a published sequence. HBV probe was designed in order to be compatible with either the RPA exonuclease III (RPA-Exo) or RPA endonuclease IV (RPA-NFO) kits (see subsequent sections for details). An internal control was developed to validate each analysis. It consisted of a double-stranded DNA (Integrated DNA Technology, Coralville, IA, USA) flanked by the forward and the reverse primers sequences, whereas the inner part is targeted by a specific control probe [33]. Sequences are described in Table 1. Primers and probes were synthetized by Kaneka Eurogentec (Seraing, Belgium).

### 2.3. Nucleic Acid Extraction

Three µL of plasma was added to 50 µL or 500 µL of 5% (*w*/*v*) Chelex 100 resin solution (Biorad, Marnes-la-Coquette, France). The internal control was added during the extraction in such a way that there were 800 copies in the final reaction mixture. After vortexing, the mixture was incubated at 56 °C for 15 min. After a second agitation, the mixture was incubated for 8 min at 95 °C. After a final stirring, the sample was briefly centrifuged in a bench-top device.

### 2.4. RPA Exo Assay–Real-Time Fluorescence Detection

The real-time RPA assay was performed in a 50 μL volume using the TwistAmp^®^ Liquid Exo kit (TwistDx, Cambridge, UK). The reaction mixture included 25 μL of 2× reaction buffer, 2.1 μL forward primer (10 µM), 2.1 μL reverse primer (10 µM), 0.6 μL probe (10 µM), 1 µL ROX 50× (ThermoFischer Scientific, Illkirch, France), 2.6 μL dH_2_O, 3.6 µL dNTPs (25 mm, ThermoFischer Scientific), 5 µL probe E mix (TwistDx), 2.5 µL of Core reaction (TwistDx), and 1 µL Exonuclease 3 (TwistDx). Extracted DNA (2 µL) was added to each tube. The addition of 2.5 μL magnesium acetate (280 mm) initiated the RPA reaction. After an incubation for 4 min at 39 °C in a thermostat C (Eppendorf, Montesson, France), the reaction was performed in a Step One Plus Applied Biosystem device (ThermoFischer Scientific) at a temperature of 39 °C.

Real-time detection was performed by quantifying the fluorescent signal ratio (FAM [6-carboxyfluorescein]/ROX [carboxyrhodamine]) every 30 s. The ROX passive reference fluorochrome was added to the reaction to weight the well-to-well signals.

### 2.5. RPA-LFA–Naked Eye Detection

The RPA NFO reaction used a modified reverse primer labeled with FAM. The sequence of the NFO probe was designed to include the internal presence of tetrahydrofurane, and a C3 blocking of the 3′ end. The HBV NFO probe was labeled with biotin and the control NFO probe was labeled with digoxigenin (Supplementary Figure S1).

The real-time RPA assay was performed in a 50 μL volume using the TwistAmp^®^ NFO kit (TwistDx). The 50 µL reaction mix included 29.5 μL rehydration buffer, 3 μL extracted DNA template, 2.1 μL FAM-labeled forward primer (10 µM), 2.1 μL reverse primer (10 µM), 0.6 µL HBV probe (10 µM), 0.6 µL control probe (10 µM), 9.8 μL dH_2_O, and 2.5 μL magnesium acetate (280 mm). The reaction was performed at 39 °C (accuracy: ± 0.5 °C) in a thermostat C (Eppendorf). The resulting HBV amplicon was dual labeled with FAM and biotin and the resulting control amplicons were dual labeled with FAM and digoxigenin.

Signals were visualized on an immunochromatographic strip (Milenia Biotech, Gießen, Germany). Biotinylated HBV amplicons were captured by streptavidin. Capture of the internal control was performed using anti-digoxigenin antibodies. Amplicons and controls were detected using gold beads coated with anti-FAM antibodies. A migration control was present at the end of the strip and consisted of antibodies directed against immunoglobulins present on the gold beads.

### 2.6. Statistics

Receiver operating characteristic (ROC) analysis was carried out with graphPad software (GraphPad Prism, San Diego, CA, USA). The measure of the area under the ROC curve (AUC) provided the performance of the assay.

## 3. Results

### 3.1. Selection of Primers and Probe and Analytical Evaluation

**Figure 1:**
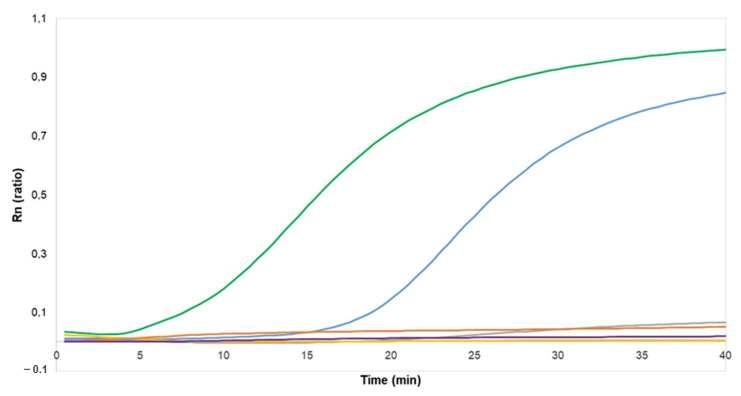
Real-time HBV RPA Exo test for primer screening. Extraction of the HBV standard sample with a viral load of 1 × 10^6^ IU/mL was carried out with 5% Chelex 100. Green line: HBV-Fc/P1R-HBV primer combination; blue: P1F-HBV/P1R_HBV; grey: HBV-Fc/HBV-R1; orange: P1F-HBV/R1-HBV; purple: non-template control; yellow: dH_2_O. Rn corresponds to the ratio of FAM (6-carboxyfluorescein) to ROX (carboxyrhodamine) fluorescence.

We evaluated four combinations of the two forward primers and two reverse primers (Table 1) for RPA Exo real-time amplification. With the combination of HBV-Fc and P1R-HBV primers, fluorescence signals were observed as quickly as 5 min after the reaction was initiated (Figure 1). The combination of P1F-HBV and P1R-HBV primers gave a positive signal after 20 min whereas the other tested combinations gave no signal.

**Figure 2:**
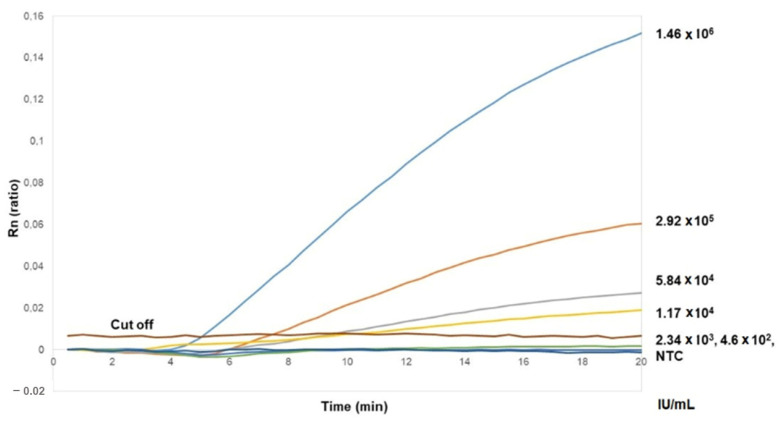
Detection limit of the HBV RPA Exo test. Amplification curves for five-fold serial dilutions ranging from viral loads of 1.46 × 10^6^ IU/mL to 4.6 × 10^2^ IU/mL. Each point of the curve represents the mean of two replicates. Rn corresponds to the ratio of FAM to ROX fluorescence. NTC refers to non-template control.

The detection limit of the combination of HBV-Fc and P1R-HBV was investigated using a panel of five-fold serial dilutions of the standard HBV sample (Figure 2). The detection limit of this real-time RPA assay was a viral load of 1.17 × 10^4^ IU/mL; taking into account the dilution factors due to the extraction and the amplification reaction, this corresponds to 1.4 IU in the reaction mixture.

### 3.2. Development of the RPA-LFA

**Figure 3:**
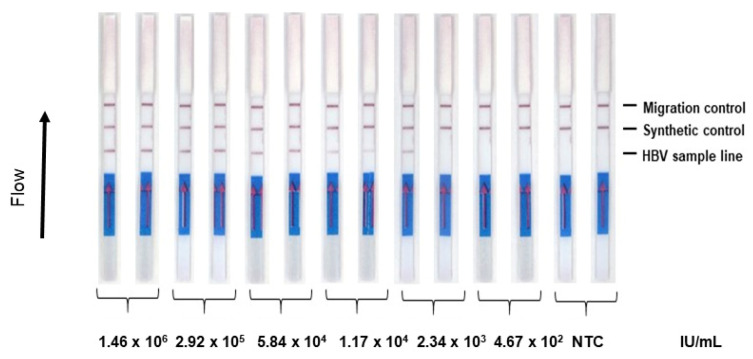
Detection limit of the HBV RPA-LFA (50 µL). To determine the detection limit of the RPA-LFA (lateral flow assay) in the format of extraction using 50 µL 5% Chelex 100, five-fold serial dilutions of the standard control ranging from viral loads of 1.46 × 10^6^ IU to 4.6 × 10^2^ IU/mL were tested. Each dilution was tested in duplicate. NTC refers to non-template control.

The detection limit of our assay was then determined on five-fold dilutions of the standard HBV sample tested in duplicate. Amplified genomes from the 1.17 × 10^4^ IU/mL (1.4 IU in the reaction tube) standard samples were detected in duplicate on strips (Figure 3).

**Figure 4:**
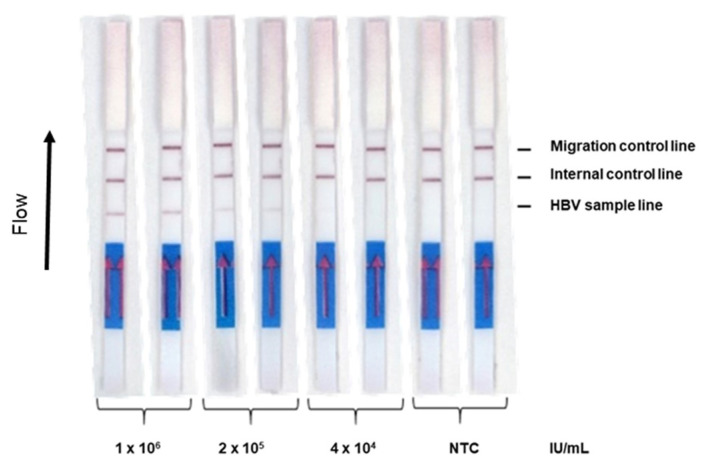
Detection limit of the HBV RPA-LFA (500 µL). To determine the detection limit of RPA-LFA in the format of extraction using 500 µL 5% Chelex 100, five-fold serial dilutions of the HBV standard control ranging from viral loads of 1 × 10^6^ IU/mL to 4 × 10^4^ IU/mL were tested. Each dilution was tested in duplicate. NTC refers to non-template control.

To obtain a detection limit of 2 × 10^5^ IU/mL, we changed our extraction conditions, using a 10 times larger volume of 5% Chelex 100. The first dilution tested was a sample with a viral load of 1 × 10^6^ IU/mL (for which the assay detected both duplicates). After a 20 min RPA reaction, the assay detected both duplicates of the 2 × 10^5^ IU/mL sample but was unable to detect either of the 4 × 10^4^ IU/mL sample duplicates (Figure 4). No additional signals were observed with RPA incubation time up to 40 min.

In all cases, the internal control was present. In addition, testing of HIV- and HCV-positive plasma samples did not result in nonspecific reactions (Supplementary Figure S2).

### 3.3. Analysis of Biological Samples

**Figure 5:**
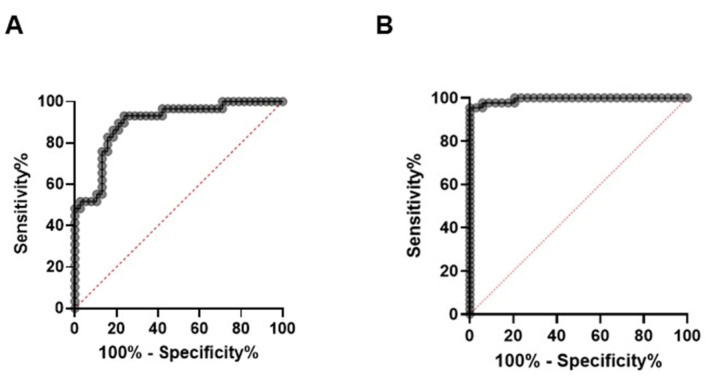
ROC (receiver operating characteristic) curve of the HBV RPA-LFA test. (**A**): 89 HBV-positive plasma samples alongside 19 HBV-negative plasma samples extracted with 50 µL 5% Chelex 100 solution, (**B**): 89 HBV-positive plasma samples alongside 19 HBV-negative plasma samples extracted with 500 µL 5% Chelex 100 solution.

In the RPA-LFA format with a threshold of 1.17 × 10^4^ IU/mL, we analyzed 89 HBV-positive samples alongside 19 HBV-negative plasma samples. The assay detected 54 out of the 60 positive samples above 1.17 × 10^4^ IU/mL and 6 out of the 29 samples below 1.17 × 10^4^ IU/mL. None of the negative samples tested positive. The AUC was 0.92 (95% CI: 0.86–0.98, *p* < 0.001) (Figure 5A). At a viral load of 1.17 × 10^4^ IU/mL, the RPA-LFA had a sensitivity of 83.7% (95% CI: 71.0–91.5%) and a specificity of 89.8% (95% CI: 79.5–95.3%).

In the RPA-LFA format with a threshold of 2 × 10^5^ IU/mL, we analyzed 89 samples (34 > 2 × 10^5^ IU/mL, 55 < 2 × 10^5^ IU/mL) alongside 19 HBV-negative plasma samples. The assay detected 33 out of the 34 samples above 2 × 10^5^ IU/mL. False-negative sample was characterized by a viral load of 2.45 × 10^5^ IU/mL. The AUC was 0.99 (95% CI: 0.99–1.00, *p* < 0.001) (Figure 5B). At a viral load of 1.98×10^5^ IU/mL, the assay showed a sensitivity of 98.6% (95% CI: 92.7–99.9%) and a specificity of 88.2% (95% CI: 73.4–95.3%). A sensitivity of 100% was achieved at a viral load of 2.52 × 10^5^ IU/mL, while a specificity of 100% was achieved at a viral load of 9.41 × 10^4^ IU/mL.

## 4. Discussion

Rapid molecular tests for the quantitative detection of HBV genomes are not available on the market. WHO recommends that in settings in which antenatal HBV DNA testing is not available, HBeAg testing can be used as an alternative to HBV DNA testing [10]. HBe antigenemia [8] has been described as an alternative marker for detecting high HBV load with a pooled sensitivity of 88.2% and a pooled specificity of 92.6% [8]. Recently, the accuracy of the Hepatitis B core-related antigen (HBcrAg) (including HBeAg, HBcAg, and P22cr) to detect samples with HBV DNA levels above 2 × 10^5^ IU/mL has been evaluated and showed an AUC of 0.94 with a sensitivity of 91.4% and specificity of 93.2% [34]. This quantitative test showed an analytical sensitivity of around 3 Log U/mL corresponding to a viral load of around 100 IU/mL [34].

The aim of our study is to develop a simple semi-quantitative nucleic acid test that focus on the 2 × 10^5^ IU/mL threshold to answer to clinical needs in order to initiate an antiviral treatment. We combined simple DNA extraction, RPA, and lateral flow immunochromatography. Then, we adapted the analytical sensitivity of our test for detecting HBV DNA to the therapeutic decision threshold, so that it can be used as a simple triage test. This combined method was able to detect viral loads at a limit of 1.17 × 10^4^ IU/mL. To fit with the viral load threshold of 2 × 10^5^ IU/mL used for initiation of MTCT prophylaxis, samples were extracted in a 10-fold larger volume. In these conditions, the analytical performance of the assay for detecting samples with a viral load above 2 × 10^5^ IU/mL gave a sensitivity of 98% and a specificity of 88% with an accuracy of 0.99. Referring to WHO ASSURED criteria for point-of-care tests in LMICS, our test is affordable; the cost is estimated to be less than USD 7. It is also specific and easy to use, having minimal steps. This test is rapid (less than 1 h), does not require specific equipment, and is deliverable.

RPA technology requires an isothermal temperature around 37 °C; this is easily obtained with simple water baths, thermos cups, or body heat [35,36]. Moreover, RPA reagents are available in lyophilized forms with high stability, facilitating their use in the field. RPA is less prone to inhibition in poorly purified DNA preparations than classical PCR amplification, thus simplifying sample preparation while reducing reagent requirements and the time-to result [26,35,37]. Hence, the extraction step was simplified for only requiring Chelex 100 resin rather than commercial kits. New developments are in progress to adapt this rapid molecular testing on whole blood samples collected with finger pricks.

The commercial availability of strips with different deposits of streptavidin and anti-digoxigenin antibodies has provided the opportunity to integrate an internal control for identification of invalid results due to the presence of inhibitors or human errors. The amplification of the internal control uses the same primers as those amplifying the HBV genome. The internal sequence of the control has been modified to differentiate the two amplified products. The complementary probe of the control DNA is tagged with digoxigenin in the 5′ end, whereas the complementary probe of HBV is tagged with biotin. Negative HBV results will only be validated if the internal control is positive. However, the presence of this control will not invalidate false-negative HBV samples with mutations that prevent them from being amplified and/or detected.

In conclusion, this proof-of-concept study demonstrates the possibility of combining a simplified rapid extraction followed by RPA amplification and detection with the naked eye on a strip including an internal control. Furthermore, we demonstrated by using the example of the MTCT diagnosis that the threshold of assay could be adjusted to the target. In-field implementation is required to further evaluate the test’s usefulness, and it needs further validation on a larger cohort of HBV-positive plasma samples from pregnant women.

## Figures and Tables

**Table 1:** Primers and probes used in this study.

	Sense	Sequence	Position *	Reference
**RPA Exo Kit: Primers and Probe**				
HBV-Fc	Forward	ATT-CGC-AGT-CCC-CAA-CCT-CCA-ATC-ACT-CAC-C	309–339	This study
HBV-R1	Reverse	AAT-ACC-ACA-TCA-TCC-ATA-TAA-CTR-AAA-GCC	755–726	Shen et al.
P1F-HBV	Forward	AAC-CTC-CAA-TCA-CTC-ACC-AAC-CTC-T	322–346	Yi et al.
P1R-HBV	Reverse	GAT-AGT-CCA-GAA-GAA-CCA-ACA-AGA-AGA	455–429	Yi et al.
EXO_HBV	Forward	CCA-AYT-TGT-CCT-GGC-TAT-CGY-TGG-ATG-[dT-FAM]-G[THF]-C[dT-BHQ1]G-CGG-CGT-TTT-ATC-AT-[Spacer C3]	353–399	This study
**RPA-NFO Kit: Primers, Probe, and Synthetic Control**				
HBV-Fc	Forward	ATT-CGC-AGT-CCC-CAA-CCT-CCA-ATC-ACT-CAC-C	309–339	This study
P1R-HBV-FAM	Reverse	FAM-GAT-AGT-CCA-GAA-GAA-CCA-ACA-AGA-AGA	455–429	Yi et al.
HBV probe	Forward	Biotin-CCA-AYT-TGT-CCT-GGC-TAT-CGY-TGG-ATG- TG[THF]-CTG-CGG-CGT-TTT-ATC-AT-[Spacer C3]	353–399	This study
Synthetic control oligonucleotide	Forward	GGCCTAAATTCGCAGTCCCCAACCTCCAATCACTT ACCAACCTCCTGTCCTCGATCATGCCCATCAGCAG CTTATGATCAATATGATCCAAACCGAGGCGCTTCC TCTTCATCCTGCTGATGCCTCATCTTCTTGTTGGT TCTTCTGGACTATCAAGGTAT		This study
Control probe	Forward	Digoxigenin-CGA-TCA-TGC-CCA-TCA-GCA-GCT-TAT-GAT- CAA-T[THF]T-GAT-CCA-AAC-CGA-GGC-G-[Spacer C3]		This study

Probe modifications: FAM: 6-carboxyfuorescein; THF: tetrahydrofuran; BHQ: black hole quencher; spacer-C3: 3′ phosphate blocker. Other abbreviations: HBV: hepatitis B virus; RPA: recombinase polymerase amplification. * Nucleotide position according to GenBank access number: LC150336, Subgenotype: A2. References: Shen et al. [29], Yi et al. [32].

## Data Availability

Not applicable.

## Supplementary Materials

The following supporting information can be downloaded at: https://www.mdpi.com/article/10.3390/diagnostics12030621/s1, Table S1: Characteristics of the Plasma samples used for Testing and Validation; Figure S1: Complete design of detection strip with test and control amplicons; Figure S2: Testing of HIV- and HCV-positive plasma samples.

## References

[1] Schweitzer A., Horn J., Mikolajczyk R.T., Krause G., Ott J.J. (2015). Estimations of worldwide prevalence of chronic hepatitis B virus infection: A systematic review of data published between 1965 and 2013. Lancet.

[2] WHO (2021). Global Progress Report on HIV, Viral Hepatitis and Sexually Transmitted Infections. https://www.who.int/publications/i/item/9789240027077.

[3] (2017). EASL 2017 Clinical Practice Guidelines on the management of hepatitis B virus infection. J. Hepatol..

[4] Stanaway J.D., Flaxman A.D., Naghavi M., Fitzmaurice C., Vos T., Abubakar I., Abu-Raddad L.J., Assadi R., Bhala N., Cowie B. (2016). The global burden of viral hepatitis from 1990 to 2013: Findings from the Global Burden of Disease Study 2013. Lancet.

[5] Pan C.Q., Duan Z., Dai E., Zhang S., Han G., Wang Y., Zhang H., Zou H., Zhu B., Zhao W. (2016). Tenofovir to Prevent Hepatitis B Transmission in Mothers with High Viral Load. N. Engl. J. Med..

[6] Jourdain G., Ngo-Giang-Huong N., Harrison L., Decker L., Khamduang W., Tierney C., Salvadori N., Cressey T.R., Sirirungsi W., Achalapong J. (2018). Tenofovir versus Placebo to Prevent Perinatal Transmission of Hepatitis B. N. Engl. J. Med..

[7] Thompson P., Morgan C.E., Ngimbi P., Mwandagalirwa K., Ravelomanana N.L.R., Tabala M., Fathy M., Kawende B., Muwonga J., Misingi P. (2021). Arresting vertical transmission of hepatitis B virus (AVERT-HBV) in pregnant women and their neonates in the Democratic Republic of the Congo: A feasibility study. Lancet Glob. Health.

[8] Boucheron P., Lu Y., Yoshida K., Zhao T., Funk A.L., Lunel-Fabiani F., Guingané A., Tuaillon E., van Holten J., Chou R. (2021). Accuracy of HBeAg to identify pregnant women at risk of transmitting hepatitis B virus to their neonates: A systematic review and meta-analysis. Lancet Infect. Dis..

[9] Funk A.L., Lu Y., Yoshida K., Zhao T., Boucheron P., van Holten J., Chou R., Bulterys M., Shimakawa Y. (2021). Efficacy and safety of antiviral prophylaxis during pregnancy to prevent mother-to-child transmission of hepatitis B virus: A systematic review and meta-analysis. Lancet Infect. Dis..

[10] WHO (2020). Prevention of Mother-to-Child Transmission of Hepatitis B Virus: Guidelines on Antiviral Prophylaxis in Pregnancy. Prevention of Mother-to-Child Transmission of Hepatitis B Virus: Guidelines on Antiviral Prophylaxis in Pregnancy.

[11] Guingané A.N., Bougouma A., Sombié R., King R., Nagot N., Meda N., Van de Perre P., Tuaillon E. (2020). Identifying gaps across the cascade of care for the prevention of HBV mother-to-child transmission in Burkina Faso: Findings from the real world. Liver Int..

[12] Belopolskaya M., Avrutin V., Kalinina O., Dmitriev A., Gusev D. (2021). Chronic hepatitis B in pregnant women: Current trends and approaches. World J. Gastroenterol..

[13] Loarec A., Nguyen A., Molfino L., Chissano M., Madeira N., Rusch B., Staderini N., Couto A., Ciglenecki I., Antabak N.T. (2022). Prevention of mother-to-child transmission of hepatitis B virus in antenatal care and maternity services, Mozambique. Bull. World Health Organ..

[14] Terrault N.A., Lok A.S.F., McMahon B.J., Chang K.M., Hwang J.P., Jonas M.M., Brown R.S., Bzowej N.H., Wong J.B. (2018). Update on prevention, diagnosis, and treatment of chronic hepatitis B: AASLD 2018 hepatitis B guidance. Hepatology.

[15] Marcuccilli F., Chevaliez S., Muller T., Colagrossi L., Abbondanza G., Beyser K., Wlassow M., Ortonne V., Perno C.F., Ciotti M. (2021). Multicenter Evaluation of the Cepheid Xpert^®^ HBV Viral Load Test. Diagnostics.

[16] Datta S., Chatterjee S., Veer V. (2014). Recent advances in molecular diagnostics of hepatitis B virus. World J. Gastroenterol..

[17] Li J., Macdonald J., von Stetten F. (2018). Review: A comprehensive summary of a decade development of the recombinase polymerase amplification. The Analyst.

[18] Mayboroda O., Katakis I., O’Sullivan C.K. (2018). Multiplexed isothermal nucleic acid amplification. Anal. Biochem..

[19] Zhao Y., Chen F., Li Q., Wang L., Fan C. (2015). Isothermal Amplification of Nucleic Acids. Chem. Rev..

[20] Craw P., Balachandran W. (2012). Isothermal nucleic acid amplification technologies for point-of-care diagnostics: A critical review. Lab Chip.

[21] Lobato I.M., O’Sullivan C.K. (2018). Recombinase polymerase amplification: Basics, applications and recent advances. Trends Anal. Chem..

[22] Piepenburg O., Williams C.H., Stemple D.L., Armes N.A. (2006). DNA detection using recombination proteins. PLoS Biol..

[23] Behrmann O., Bachmann I., Spiegel M., Schramm M., Abd El Wahed A., Dobler G., Dame G., Hufert F.T. (2020). Rapid Detection of SARS-CoV-2 by Low Volume Real-Time Single Tube Reverse Transcription Recombinase Polymerase Amplification Using an Exo Probe with an Internally Linked Quencher (Exo-IQ). Clin. Chem..

[24] Cherkaoui D., Huang D., Miller B.S., Turbé V., McKendry R.A. (2021). Harnessing recombinase polymerase amplification for rapid multi-gene detection of SARS-CoV-2 in resource-limited settings. Biosens. Bioelectron..

[25] Xia S., Chen X. (2020). Single-copy sensitive, field-deployable, and simultaneous dual-gene detection of SARS-CoV-2 RNA via modified RT-RPA. Cell Discov..

[26] Boyle D.S., Lehman D.A., Lillis L., Peterson D., Singhal M., Armes N., Parker M., Piepenburg O., Overbaugh J. (2013). Rapid detection of HIV-1 proviral DNA for early infant diagnosis using recombinase polymerase amplification. mBio.

[27] Bai X., Ma X., Li M., Li X., Fan G., Zhang R., Wang R., Duan Q., Shen X., Xie Y. (2020). Field applicable detection of hepatitis B virus using internal controlled duplex recombinase-aided amplification assay and lateral flow dipstick assay. J. Med. Virol..

[28] Zhang B., Zhu Z., Li F., Xie X., Ding A. (2021). Rapid and sensitive detection of hepatitis B virus by lateral flow recombinase polymerase amplification assay. J. Virol. Methods.

[29] Shen X.X., Qiu F.Z., Shen L.P., Yan T.F., Zhao M.C., Qi J.J., Chen C., Zhao L., Wang L., Feng Z.S. (2019). A rapid and sensitive recombinase aided amplification assay to detect hepatitis B virus without DNA extraction. BMC Infect. Dis..

[30] Ichzan A.M., Hwang S.H., Cho H., Fang C.S., Park S., Kim G., Kim J., Nandhakumar P., Yu B., Jon S. (2021). Solid-phase recombinase polymerase amplification using an extremely low concentration of a solution primer for sensitive electrochemical detection of hepatitis B viral DNA. Biosens. Bioelectron..

[31] Vanhomwegen J., Kwasiborski A., Diop A., Boizeau L., Hoinard D., Vray M., Bercion R., Ndiaye B., Dublineau A., Michiyuki S. (2021). Development and clinical validation of loop-mediated isothermal amplification (LAMP) assay to diagnose high HBV DNA levels in resource-limited settings. Clin. Microbiol. Infect..

[32] Yi T.T., Zhang H.Y., Liang H., Gong G.Z., Cai Y. (2020). Betaine-assisted recombinase polymerase assay for rapid hepatitis B virus detection. Biotechnol. Appl. Biochem..

[33] Higgins O., Clancy E., Forrest M.S., Piepenburg O., Cormican M., Boo T.W., O’Sullivan N., McGuinness C., Cafferty D., Cunney R. (2018). Duplex recombinase polymerase amplification assays incorporating competitive internal controls for bacterial meningitis detection. Anal. Biochem..

[34] Shimakawa Y., Ndow G., Njie R., Njai H.F., Takahashi K., Akbar S.M.F., Cohen D., Nayagam S., Jeng A., Ceesay A. (2020). Hepatitis B Core-related Antigen: An Alternative to Hepatitis B Virus DNA to Assess Treatment Eligibility in Africa. Clin. Infect. Dis. Off. Publ. Infect. Dis. Soc. Am..

[35] Lillis L., Lehman D., Singhal M.C., Cantera J., Singleton J., Labarre P., Toyama A., Piepenburg O., Parker M., Wood R. (2014). Non-instrumented incubation of a recombinase polymerase amplification assay for the rapid and sensitive detection of proviral HIV-1 DNA. PLoS ONE.

[36] Crannell Z.A., Rohrman B., Richards-Kortum R. (2014). Equipment-free incubation of recombinase polymerase amplification reactions using body heat. PLoS ONE.

[37] Lillis L., Siverson J., Lee A., Cantera J., Parker M., Piepenburg O., Lehman D.A., Boyle D.S. (2016). Factors influencing Recombinase polymerase amplification (RPA) assay outcomes at point of care. Mol. Cell. Probes.

