# Supplementary material for: Rapid Diagnostic Test for Hepatitis B Virus Viral Load Based on Recombinase Polymerase Amplification Combined with a Lateral Flow Read-Out
Source: Diagnostics (Basel). 2022 Mar 2;12(3):621. doi: 10.3390/diagnostics12030621 (PMC8946908; doi:10.3390/diagnostics12030621)

Table S1. Characteristics of the Plasma samples used for Testing and Validation. ND: Not determined; +: Positive; - : Negative.

Sample	Genotype	Viral Load	Results	
			Extraction 50 µL	Extraction 500 µL
E00117	A	> 1.1 10e8	+	+
E06195	A	> 1.1 10e8	+	+
E14176	B	> 1.1 10e8	+	+
E1812-	B	> 1.1 10e8	+	+
E45845	D	> 1.1 10e8	+	+
E49881	C	> 1.1 10e8	+	+
E55133	C	> 1.1 10e8	+	+
E61695	B	> 1.1 10e8	+	+
E62174	A	> 1.1 10e8	+	+
E80746	B	> 1.1 10e8	+	+
E94093	C	> 1.1 10e8	+	+
E98559	C	26654000	+	+
E69247	E	21023350	+	+
E97194	C	13365400	+	+
E76741	A	7645489	+	+
E24085	E	6689030	+	+
E3153-	A	6150761	+	+
E20215	D	4987748	+	+
E09428	ND	4970052	+	+
E29222	A	2347600	+	+
E15269	D	1464222	+	+
E25058	A	1114920	+	+
E65547	A	1030442	+	+
E80844	D	573938	+	+
E4211-	E	383353	+	+
E06118	B	300783	+	+
E84470	E	260079	+	+
E17840	C	245678	+	-
E89788	D	226830	+	+
E27469	E	214840	+	+
E86382	D	200468	+	+
E09151	A	196469	+	+
E15513	D	163109	+	+
E82183	A	153760	-	-
E73910	D	152678	+	+
E51677	D	108540	+	+
E4074-	E	79732	+	-
E83094	D	78493	+	-
E55919	A	72393	+	-
E56722	D	68150	+	-
E29210	A	65469	+	-
E14413	E	57220	+	-
E53843	D	53357	+	-
E4875-	E	43153	+	-
E08444	D	40963	+	-

E49523	A	39610	-	-
E56666	D	39450	+	-
E02248	D	38065	+	-
E16088	B	37871	+	-
E87806	A	36099	+	-
E31462	A	36054	+	-
E79349	E	33586	+	-
E27550	D	32794	+	-
E96604	A	32006	-	-
E24437	A	31115	+	-
E94736	B	31085	-	-
E26819	D	29791	+	-
E58230	D	27711	-	-
E70297	A	26272	+	-
E00863	A	23413	-	-
E42606	B	13283	-	-
E45424	D	9776	+	-
E01565	D	9487	-	-
E81738	B	9315	-	-
E32460	A	9269	-	-
E40219	A	9260	-	-
E28308	D	9130	-	-
E29434	E	9100	-	-
E04224	A	8927	+	-
E54099	C	8706	-	-
E85356	D	8595	+	-
E6494-	D	8427	+	-
E68958	A	7609	+	-
E22411	A	7115	-	-
E70977	D	6783	+	-
E44177	E	6233	-	-
E96998	D	3100	-	-
E00627	D	836	-	-
E80955	A	752	-	-
E79047	D	703	-	-
E47628	B	696	-	-
E66533	F	624	-	-
E88834	D	258	-	-
E05446	D	246	-	-
E61960	D	245	-	-
E53798	A	211	-	-
E14324	A	149	-	-
E29362	B	112	-	-
E31178	D	18	-	-

Figure S1. Complete design of detection strip with test and control amplicons. Ab: Antibodies, Dig: Digoxigenin, Biot: Biotin.

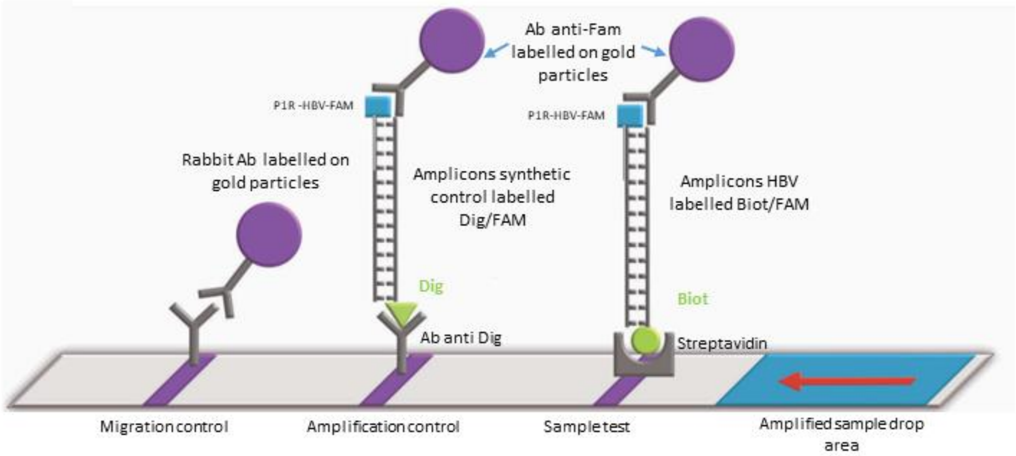

Figure S2. Testing of HIV- and HCV – positive samples. Samples were analyzed with the HBV-RPA LFA test. NTC refers to non-template control.

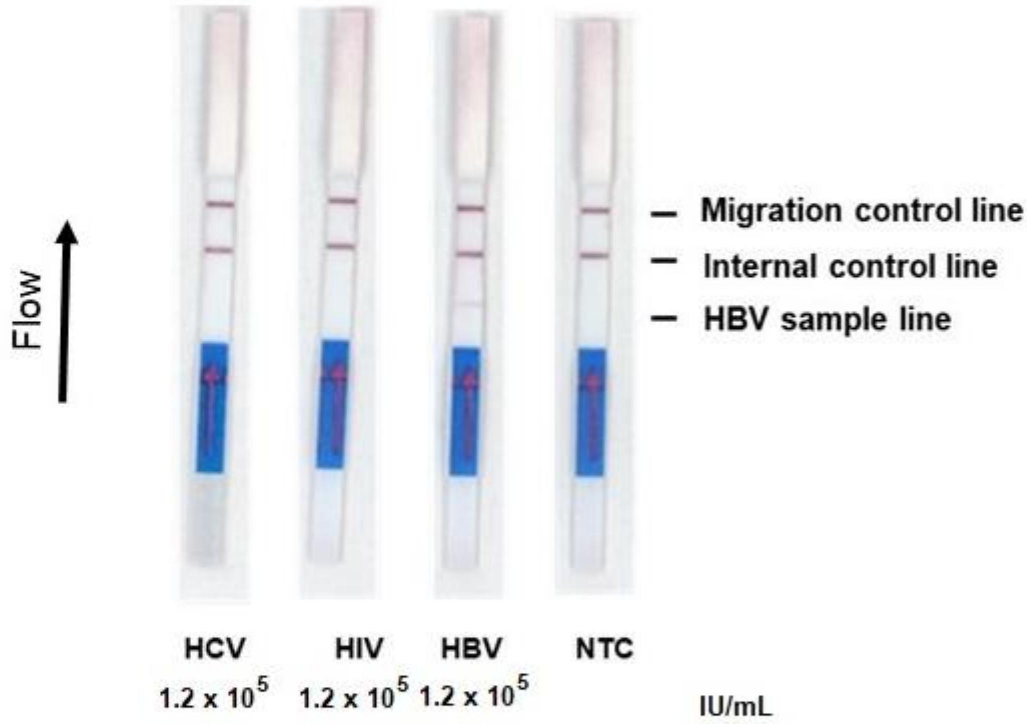

Supplement: Supplementary file 1 [file diagnostics-12-00621-s001.zip › diagnostics-1568232-supplementary.pdf]
